# Axon zippering in neuronal cell culture and its biophysical modeling

**DOI:** 10.1186/1471-2202-16-S1-P298

**Published:** 2015-12-18

**Authors:** Daniel Smit, Coralie Fouquet, Frederic Pincet, Alain Trembleau, Martin Zapotocky

**Affiliations:** 1 Institute of Physiology of the Czech Academy of Sciences, Prague, Czech Republic; 2 Institute of Biophysics and Informatics, First Faculty of Medicine, Charles University in Prague, Prague, Czech Republic; 3 IBPS, Neuroscience Paris Seine, CNRS UMR8246, Inserm U1130, UPMC UM 119, Université Pierre et Marie Curie, Paris, France; 4 Laboratoire de Physique Statistique, Ecole Normale Superieure, Paris, France

## 

During neural development, the growing axons frequently adhere to each other and form tight bundles. This process contributes to the setting up of correct connectivity in, e.g., the mammalian olfactory system [1]. Here we report an experimental and theoretical investigation of axon bundling in olfactory epithelium explants; in this system, the dynamics of axon-axon interactions can be directly examined.

Our analysis is based on time-lapse optical microscopy observations of axon growth in primary cell culture. Olfactory epithelium explants from mouse embryos (day 13-14) were cultured on laminin substrate for two days and then recorded using DIC videomicroscopy for up to 24 hours. The growing axons established a dense network within which large bundles of axons were progressively formed. This dynamics was driven by numerous fast "zippering" processes, during which the length of the segment along which two axons adhere to each other was increased. Similar processes were inferred in 1982 from electron-microscopy-based analysis of sensory neurites in Xenopus embryos [2], but have been very rarely studied in the subsequent literature.

We digitized a collection of 30 spontaneous zippering processes observed in the developing axon network. During zippering, the vertex (i.e. the point where the two axons meet) advances with a typical velocity of 0.2 to 1 µm/min. The distance between immediate position of the vertex and the final equilibrium position decreases either linearly with sudden arrest or exponentially in time. We observed similar behaviour, at velocities of 1 to 3 µm/min, for zippering events induced during micro-manipulation experiments.

To explain these observations, we developed a simplified biophysical model that includes the adhesive force between the axons and the mechanical tension in the axons, as well as adhesion and anisotropic friction with the substrate. A static zipper is obtained if the tensile and adhesive forces are balanced. The model predicts that the linear regime of convergence to equilibrium can arise from a perpendicular outer force acting on one of the axons, while the exponential regime is a result of sudden change in axial tension of the axon. The parameters of the model were estimated from calibrated micro-manipulations of the culture, using a biotinylated red blood cell as force transducer [3]. These experiments allowed us to measure the tension within the axon shaft (typically about 1 nN) and deduce the force of adhesion between two axon shafts (of order 100 pN).

To account for additional factors, we implemented a molecular-dynamics-type simulation. The beads-and-springs representation of each axon permits to include bending rigidity and allows for an inhomogeneous distribution of tension along the axon. To implement the interaction between axons, we adapted results from contact mechanics [4] for cylindrical bodies. This permits to avoid artifacts that would result from direct adhesive interaction between beads. We compare the dynamics of zippering in this detailed model to the predictions of the simplified analytical model.
